# Characterization of the mitochondrial genome of *Microphysogobio tungtingensis* (Cypriniformes) and its taxonomic status

**DOI:** 10.1080/23802359.2020.1763215

**Published:** 2020-05-18

**Authors:** Huahui Luo, Zhaoyu Wei, Yongguang Xie, Huaixin Wang, Hong Yuan, Dunxue Chen

**Affiliations:** a College of Animal Science, Guizhou University, Guiyang, P.R. China; b Zunyi Academy of Agricultural Sciences, Zunyi, P.R. China; c Research Center of Fishery Resources and Environment, Guizhou University, Guiyang, P.R. China

**Keywords:** *Microphysogobio tungtingensis*, mitogenome, arrangement, phylogenetic analysis

## Abstract

In the present study, the complete mitochondrial genome of *Microphysogobio tungtingensis* has been amplified with 16 pairs of primers. There are 16 627 base pairs has been identified and deposited in the GenBank with accession numbers MN970213. The arrangement was similar to typical vertebrate mitochondrial, including 13 protein-coding genes, 22 transfer RNAs, 2 ribosomal RNAs genes and a noncoding control region. The overall base composition of *M. tungtingensis* was G + C: 42.9%, A + T: 57.1%, apparently with a slight AT bias. Phylogenetic analysis showed that *M. tungtingensis* was close to *M. fukiensis*.

Twenty species of *Microphysogobio* have been found in China (Huang et al. 2018) while 30 species were identified in the world (Huang et al. 2017). *Microphysogobio tungtingensis* is a genus of small benthic gudgeons with 60 mm in length, which is widely distributed in the Dongting Lake and other streams. There are significant genetic divergence between *Microphysogobio* (Huang et al. 2017), further study of the taxonomic status among *Microphysogobio* is necessary with more species. For the above-mentioned purpose, *Microphysogobio tungtingensis* was obtained from the Wuyang River (N108.15, E27.04) and soaked with ethyl alcohol (95%) in Guizhou University. The total genomic DNA was extracted from skeletal muscle tissues of the fishes with the DNA extraction Kit (Chen et al. 2012) (Invitrogen, Carlsbad, CA).

The mitogenome had the typical vertebrate mitochondrial gene arrangement, including 13 protein-coding genes, 22 transfer RNAs, 2 ribosomal RNAs genes, and a noncoding control region (CR) (Yang et al. 2016). CR of 927 bp length is located between tRNA^Pro^ and tRNA^Phe^. 22 tRNAs are interspersed between ribosomal RNA and protein-coding genes and range in size from 65 to 80 nucleotides.

In order to reassess the taxonomic status of *Microphysogobio*, 12 species were chosen. Phylogenetic analyses were constructed by the neighbor-joining (NJ) method performed on total mitogenomes (Zou et al. 2017) (Figure 1).

**Figure 1.**
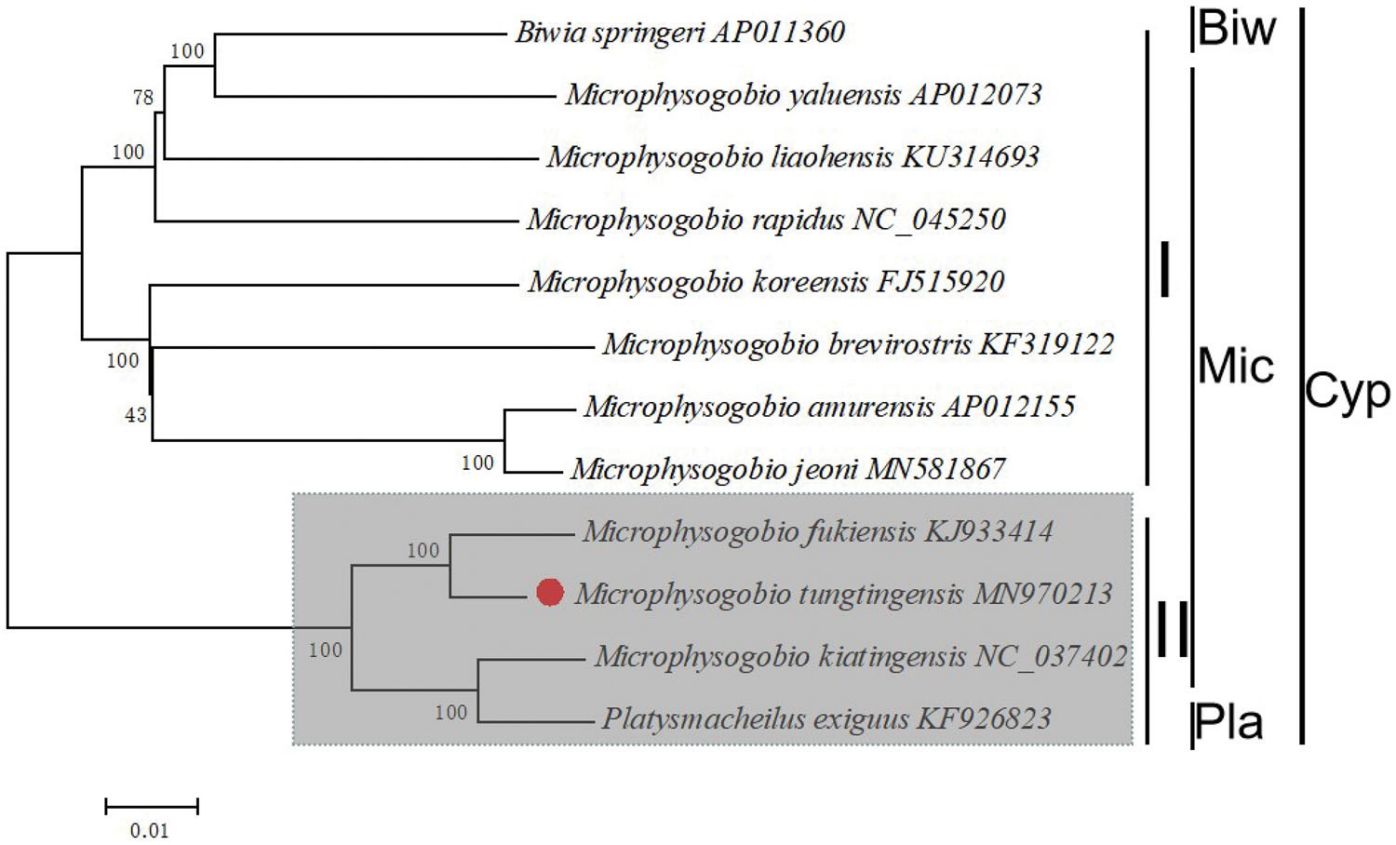
The phylogenetic analyses investigated using N-J analysis indicated evolutionary relationships among 12 species based on total mitogenomes. Biw: Biwia, Mic: Microphysogobio, Pla: Platysmacheilus, Cyp:Cyprinidae.

The phylogenetic tree revealed that the *Microphysogobio* could be divided into two clades (I and II). In group I, there were 8 species clustered together, while the group II with 4. However, the out group *Biwia springeri* clustal together with group, *Platysmacheilus exiguus* is sister to *M. koreensis* which were contrary to expectations. Our work confirmed that the *M. yaluensis* was close to *B. springeri* which is accordance with previously reported (Park et al. 2016 (Yang et al., 2016) ). The *M. tungtingensis* and *M. fukiensis* showed a closest phylogenetic relationship, then clustered with *M. kiatingensis.*

## Data Availability

The data that support the findings of this study are available in National Center for Biotechnology Information（https://www.ncbi.nlm.nih.gov/）with accession number MN970213.
